# Transformed astrocytes confer temozolomide resistance on glioblastoma via delivering ALKBH7 to enhance APNG expression after educating by glioblastoma stem cells‐derived exosomes

**DOI:** 10.1111/cns.14599

**Published:** 2024-02-08

**Authors:** Xinglei Liu, Liang Liu, Anyi Wu, Shilu Huang, Zhipeng Xu, Xiaopei Zhang, Zengyang Li, Haoran Li, Jun Dong

**Affiliations:** ^1^ Department of Neurosurgery The Second Affiliated Hospital of Soochow University Suzhou China; ^2^ Department of Neurosurgery, Affiliated Nanjing Brain Hospital Nanjing Medical University Nanjing Jiangsu China

**Keywords:** ALKBH7, APNG, glioblastoma, glioblastoma stem cells‐derived exosomes, TMZ resistance, tumor‐associated astrocytes

## Abstract

**Background:**

Glioblastoma is the most malignant primary brain tumor in adults. Temozolomide (TMZ) stands for the first‐line chemotherapeutic agent against glioblastoma. Nevertheless, the therapeutic efficacy of TMZ appears to be remarkably limited, because of low cytotoxic efficiency against glioblastoma. Besides, various mechanical studies and the corresponding strategies fail to enhancing TMZ curative effect in clinical practice. Our previous studies have disclosed remodeling of glial cells by GSCs, but the roles of these transformed cells on promoting TMZ resistance have never been explored.

**Methods:**

Exosomes were extracted from GSCs culture through standard centrifugation procedures, which can activate transformation of normal human astrocytes (NHAs) totumor‐associated astrocytes (TAAs) for 3 days through detect the level of TGF‐β, CD44 and tenascin‐C. The secretive protein level of ALKBH7 of TAAs was determined by ELISA kit. The protein level of APNG and ALKBH7 of GBM cells were determined by Western blot. Cell‐based assays of ALKBH7 and APNG triggered drug resistance were performed through flow cytometric assay, Western blotting and colony formation assay respectively. A xenograft tumor model was applied to investigate the function of ALKBH7 in vivo. Finally, the effect of the ALKBH7/APNG signaling on TMZ resistance were evaluated by functional experiments.

**Results:**

Exosomes derived from GSCs can activate transformation of normal human astrocytes (NHAs)to tumor‐associated astrocytes (TAAs), as well as up‐regulation of ALKBH7expression in TAAs. Besides, TAAs derived ALKBH7 can regulate APNG gene expression of GBM cells. After co‐culturing with TAAs for 5 days, ALKBH7 and APNG expression in GBM cells were elevated. Furthermore, Knocking‐down of APNG increased the inhibitory effect of TMZ on GBM cells survival.

**Conclusion:**

The present study illustrated a new mechanism of glioblastoma resistance to TMZ, which based on GSCs‐exo educated TAAs delivering ALKBH7 to enhance APNG expression of GBM cells, which implied that targeting on ALKBH7/APNG regulation network may provide a new strategy of enhancing TMZ therapeutic effects against glioblastoma.

## Graphical abstract

ALKBH7 was upregulated in TAAs, which were stimulated by GSC‐derived exosomes. Next, ALKBH7 can be transported into GBM cells and lead to upregulation of APNG. These results further illustrated the mechanism of the ALKBH7/APNG axis contributing to TMZ resistance.

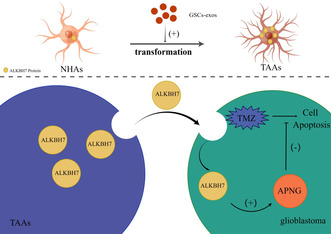

## 1 INTRODUCTION

Characterized by high invasiveness, high therapeutic resistance, high recurrence, and dismal prognosis, glioblastoma comprised the predominant refractory primary brain tumor in adults.^1^, ^2^, ^3^ Temozolomide (TMZ) can readily penetrate the blood–brain barrier and be prescribed as the first‐line chemotherapeutic agent against glioblastoma in the current comprehensive therapies. However, resistance to TMZ is still unavoidable in an overwhelming majority of glioblastoma patients,^4^, ^5^ Nearly, all preclinical studies failed to overcome TMZ resistance to superior clinical therapeutic efficacy up to now. Consequently, further exploring of new potential mechanisms of TMZ resistance is worthy for in‐depth study.

Glioblastoma stem cells (GSCs) have been demonstrated to play vital roles in oncogenesis, malignant progression, formation of highly immune‐suppressive tumor microenvironment (TME), posttherapeutic recurrence, and high resistance to current standard therapies.^6^ Previous studies have documented that drug resistance against TMZ is multifactorial, engaging not only the intracellular processes but also elements within the glioblastoma microenvironment,^7^ and TME participates in tumor proliferation and substantially modifies treatment responses and clinical outcomes.^8^, ^9^ Glial cells, especially astrocytes are essential components of constructing glioblastoma microenvironment. The mutual interactions between astrocytes and tumor cells form a complex communication network, which profoundly affects chemo/radio‐resistance.^10^ Previous research has revealed that exosomes released from GSCs can modulate the stromal cells of TME to remodel an optimized environment favoring glioblastoma development.^11^, ^12^, ^13^ Besides, our previous studies have disclosed that GSCs can induce malignant transformation of various stromal cells in TME after direct mutual interactions between them in a dual color tracing model, including glial cells, mesenchymal stem cells, dendritic cells, etc.^14^, ^15^, ^16^ The crosstalk between GSCs and macrophages via GSCs‐derived exosomes (GSCs‐exos) have been explored as well, which resulted in enhanced pro‐tumor effects of tumor‐associated macrophages (TAMs).^17^ However, whether GSCs‐exos‐educated astrocytes can transform into tumor‐associated astrocytes (TAAs), and enhance the chemo‐resistance of glioblastoma has not been investigated.

## 2 MATERIALS AND METHODS

### 2.1 Cell culture

Human glioblastoma cell lines SNB19 and SF295 cells (American Type Culture Collection, ATCC) were cultured in a DMEM medium (Gibco, USA) supplemented with 10% fetal bovine serum (FBS, Gibco, USA). Normal human astrocytes (NHAs) (ScienCell, Carlsbad, CA, USA) were grown in a DMEM medium supplemented with 10% FBS and 1% astrocyte growth supplement (ScienCell, USA). Human GSCs cell lines GSC11 and GSC23 (MD Anderson Cancer Center, USA) were cultured in a DMEM/F12 medium (Gibco, USA) supplemented with B27 (1×, Gibco, USA), EGF (20 ng/mL) (Thermo Scientific, USA), and b‐FGF (20 ng/mL) (Thermo Scientific, USA). All cells were maintained in a cell incubator at 37°C under humidified atmosphere containing 5% CO_2_.

### 2.2 Exosomes isolation and analysis

Exosomes were extracted from the cell culture medium of GSC11 or GSC23 cells through multiple centrifugation procedures with VEX™ Exosome Isolation Reagent (Vazyme, China). Briefly, the collected culture medium was centrifuged at 2000 *g* for 30 min, then 12,000 *g* for 45 min to remove cell debris and large vesicles. For exosome purification, the supernatant was ultracentrifuged at 100,000 *g* for 60 min at 4°C to collect the pellet, then resuspended in 50–100 μL PBS for the subsequent studies. The protein content of exosomes was detected with MicroBCA protein assay (Thermo Scientific, USA). The positive exosomal markers, TSG101 and HSP70, and a negative marker Calnexin (Abcam, UK) were applied in Western blotting for identifying the purified GSCs‐derived exosomes.

### 2.3 Noncontact cell coculture of transformed astrocytes and glioblastoma cells

Transwell‐6 system (Corning Inc, USA) was applied to analyze the indirect interactions between glioblastoma cells and TAAs. SNB19 or SF295 cells were plated in 0.4 μm porous transwell inserts (Corning Inc, USA) suspended over TAAs plated at a 1:4 ratio, then co‐cultured in DMEM with 10% FBS for 5 days.

### 2.4 siRNA transfection

siRNA‐mate was used for transient transfection of the corresponding siRNAs into SNB19 or SF295 cells. For siRNA transfection, cells were plated in a six‐well plate (2000 μL medium/well) and cultured overnight in a cell incubator, followed by transfection with the target siRNAs or the control siRNAs for another 48 h. Knockdown efficiency of the target genes was confirmed by qPCR.

### 2.5 Annexin V‐FITC/PI cell apoptosis detection

SNB19 or SF295 cells were co‐cultured with or without TAAs for 5 days, then were treated with TMZ (Sigma Aldrich, USA) for subsequent 48 h (300 μM for SNB19 cells, 200 μM for SF295 cells, according to IC50 value of each cell). Cell apoptosis was evaluated with Annexin V‐FITC/PI Apoptosis Kit I (MULTI Sciences, China). A total of 1 × 10^6^ cells were collected from each group and stained with FITC and PI following the manufacturer's protocols.

### 2.6 Quantification of ALKBH7 level by ELISA

A 100 μL culture medium was collected from NHAs or TAAs, and the AlkB homolog 7 (ALKBH7) protein content was determined with ALKBH7 ELISA kit (MEIBIAO BIOLOGY, China) following the manufacturer's instructions.

### 2.7 RNA extraction and qRT‐PCR

Total RNA was extracted from cells with TRIzol (Sigma‐Aldrich, USA), followed by cDNA synthesis through reverse transcription cDNA Kit (Thermo‐Fisher, USA) according to the manufacturer's instructions. SYBR PrimeScript RT‐PCR Kit (Novoprotein, China) was applied to detect the PCR amplification products. The qPCR data were analyzed using the 2^−ΔΔCt^ method. Total RNA level was normalized with GAPDH. The primers used in this study were designed and synthesized by Sangon Biotech (China).

### 2.8 Western blotting

Cells were harvested and lysed with standard PLC lysis buffer containing protease and phosphatase inhibitors (Sigma‐Aldrich, USA). Then the lysate was centrifuged to collect supernatant. Protein concentration was determined using BCA assay (Thermo‐Fisher Scientific, USA). Finally, equivalent amounts of protein (30 μg) were separated with 7.5%–12.5% SDS‐PAGE gel with electrophoresis, then were electro‐transferred onto PVDF membrane (NEN Research Products, USA). Membrane was soaked in 5% nonfat milk for 2 h at room temperature. The primary antibodies were applied at dilutions of 1:1000 for anti‐ALKBH7 (A2331, ABclonal, China), anti‐Alkylpurine‐DNA‐N‐glycosylase (APNG) (ab155092, Abcam, UK), MGMT (Proteintech, 17195‐1‐AP, China), or anti‐γ‐H2AX (97148SF, CST, USA), respectively, at 4°C overnight. After incubation with the primary antibody, the membrane was washed three times with PBS with 0.1% Tween 20 for 10 min, then incubated with the second antibody specific for the primary antibody (BioRad Laboratories, USA). The binding was visualized with Chemiluminescence Reagent Plus (PerkinElmer, USA) and analyzed in ImageJ.

### 2.9 Cell viability analysis

Cell viability was evaluated with CCK‐8 assay. Briefly, cells were seeded at a density of 1000 cells/well in 96‐well plate and incubated in a 5% CO_2_ incubator at 37°C overnight. Then TMZ (final concentration varying from 0 to 1200 μM with 200 μM interval) was administered in each well for 48 h. Ten microliters CCK‐8 solution (Meilunbio, China) were added to each well and incubated for 2 h at 37°C. Last, absorbance (OD value) was detected at a wavelength of 450 nm. The “log (inhibitor) versus normalized slope of response variable” method was applied to calculate the 50% inhibition concentration (IC50) of TMZ with GraphPad Prism 8.0 (GraphPad Software, USA). In the subsequent experiments associated with TMZ administration, 1/2 of TMZ IC50 value was applied in the corresponding cell culture.

### 2.10 Colony formation assay

For colony formation assay, a six‐well plate was seeded with 1000 cells per well and treated with TMZ for 48 h. After 15 days of growth, the resulting colonies were washed twice with PBS, fixed with 4% paraformaldehyde for 30 min, and stained with 0.1% crystal violet for 30 min.

### 2.11 Lentivirus infection

SNB19 cells were stably infected with lentiviruses carrying sh‐ALKBH7 or negative control (NC) to generate SNB19‐shALKBH7, SNB19‐shNC cells, respectively. Transfection of the expression plasmids of ALKBH7 in SNB19 cells was performed with Lipofectamine 3000 kit (Invitrogen, Carlsbad, CA, USA) according to the manufacturer's instructions.

### 2.12 In vivo xenograft model

All animal experiments were conducted in accordance with the ethical standards of the animal care and use committee of Soochow University (SUDA 20210708A03). Ectopic xenograft model was prepared by subcutaneous injection of 1 × 10^6^ SNB19 cells (sh‐NC or sh‐ALKBH7) into the right armpit of 4‐week‐old female Balb/c nude mice (4 mice per group). The mice were divided randomly into four groups, then treated with TMZ via intraperitoneal injection (66 mg/kg per day for 5 days) or the same dose of DMSO as a control, 2 weeks after tumor cells inoculation. The tumor volume was measured and calculated by the formula v(mm^3^) = length × width^2^ × 0.5. The mice were sacrificed 6 weeks later under general anesthesia, and the xenografts were harvested. For the evaluation of intracranial glioblastoma growth, the orthotopic model was established. Briefly, a suspension of 1 × 10^5^ SNB19 sh‐NC or sh‐ALKBH7 cells in 10 μL DMEM was stereotactically injected into the right caudate nucleus of Balb/c nude mice (randomly assigned into 3 mice/group). After 14 days of implantation, TMZ (temozolomide) was administered intraperitoneally at a dosage of 66 mg/kg per day for consecutive 5 days. When neurological symptoms appeared obviously, mice were euthanized, then brains were sampled and fixed in 4% paraformaldehyde, followed by paraffin embedding and sectioning for hematoxylin and eosin staining.

### 2.13 Statistical analysis

All statistical analyses were conducted with GraphPad software version 8.0 (GraphPad Software, San Diego, CA, USA). Results were presented as means ± SD standard deviation on three independent experiments. The normality of data distribution was analyzed by the Shapiro–Wilk test. Student's *t* test was performed to analyze the statistical difference between two groups, and analysis of variance (ANOVA) was applied to evaluate the differences between multiple groups. The *p*‐value < 0.05 was considered statistically significant (*p < 0.05, **p < 0.01, ***p < 0.001, ****p < 0.0001). *p*‐value > 0.05 was considered not significant and was denoted by “NS.”

## 3 RESULTS

### 3.1 GSCs‐exos educated astrocytes harbored TAAs‐like phenotypes

**FIGURE 1:**
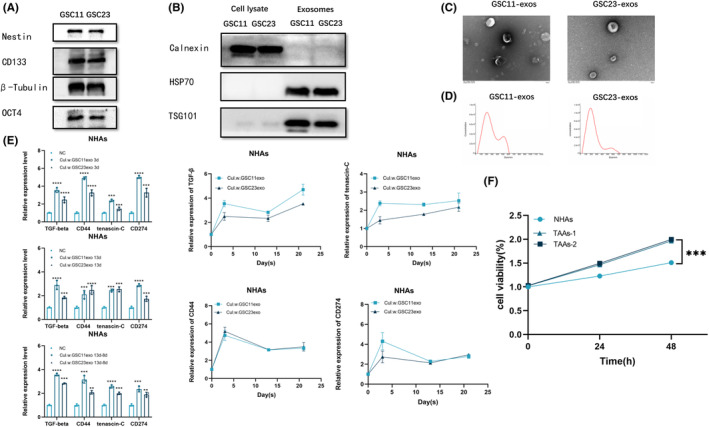
Transformation of NHAs into TAAs under GSCs‐exos education. (A) Expression of GSCs markers, Nestin, CD133, and OCT4 in GSC11 and GSC23 cells. (B) Exosomes purified from the cell culture medium of GSCs. Expression of the positive markers of exosomes TSG101 and HSP70, as well as the negative marker calnexin, were detected by Western blot, and expression of these three markers was evaluated in the cell lysate of GSCs as well. (C) Transmission electron microscopy of exosomes derived from GSCs. Scale bars: 100 nm. (D) Size distribution analysis of GSCs‐derived exosomes. (E) QRT‐PCR analysis on TGF‐β, CD44, tenascin‐C, and CD274 expression in TAAs and NHAs. (F) TAAs had obvious morphological changes with enhanced proliferation. **p < 0.01, ***p < 0.001, and ****p < 0.0001.

Cell surface markers of glioblastoma stem‐like cells (Nestin, CD133, and OCT4) were positive for both GSC11 and GSC23 cells (Figure 1A). To explore whether NHAs engulf GSCs‐derived exosomes, the exosomes harvested from the supernatant of culture medium of GSC11 and GSC23 cells were purified and analyzed with Western blot, transmission electron microscopy (TEM), and nanoparticle tracking analysis (NTA), respectively, which disclosed positive expression of exosomes marker proteins TSG101 and HSP70, while calnexin was absent in exosomes and only present in the lysate of GSCs (Figure 1B). Besides, a typical lipid bilayer membrane can be observed in the purified exosomes, and the size of exosomes was in accord with the standard range confirmed by both TEM and NTA (Figure 1C,D).

GSC11 or GSC23 cells‐derived exosomes were supplemented with a culture medium of NHAs (40 μg/mL) every 3 days. The expression level of TGF‐β, CD44, CD274, and tenascin‐C in NHAs increased significantly at day 3, kept at a stable high level till day 13, and did not decrease since day 13 (beginning of no GSCs‐exosomes addition) until day 21 (end of observation) (Figure 1E). Compared with NHAs, TAAs showed enhanced proliferation (Figure 1F) and had obvious morphological changes with larger cell bodies, more and longer processes (Figure S1A).

### 3.2 Upregulation of APNG expression conferred TMZ resistance to glioblastoma cells after co‐culturing with TAAs

**FIGURE 2:**
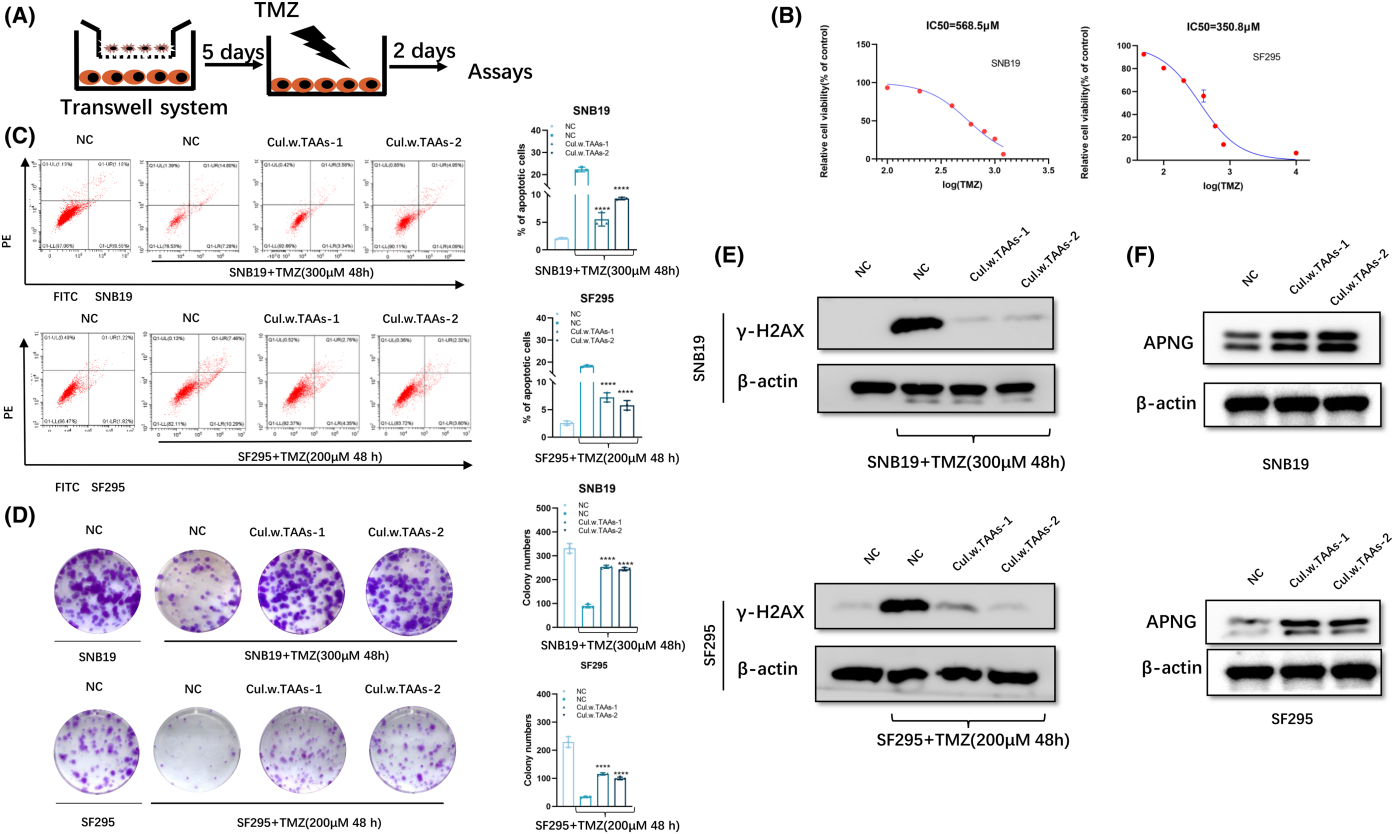
APNG overexpression conferred TMZ resistance onto glioblastoma cells. (A) Schematics of glioblastoma cells/TAAs indirect co‐culture system. (B) The IC50 values of TMZ by CCK‐8 assay in SNB19 and SF295 cells. (C) SNB19 and SF295 cells were co‐cultured with TAAs for 5 days, then were exposed to TMZ for 48 h, followed by apoptosis analysis by flow cytometry. (D) After co‐culturing with TAAs, SNB19, and SF295, cells were treated with TMZ (300 μM for SNB19, 200 μM for SF295) for 48 h, then a colony formation assay was performed. Tumor cells under TMZ exposure alone without co‐culturing with TAAs were set as a control. (E) Western blot analysis of γ‐H2AX expression in SNB19 or SF295cells after culturing with TAAs for 5 days, then treated with TMZ (300 μM for SNB19, 200 μM for SF295) 48 h. β‐actin was set as the loading control. SNB19 and SF295 cells under TMZ exposure alone without co‐culturing with TAAs served as a control. (F) Protein levels of APNG in glioblastoma cells co‐cultured with TAAs were assayed with Western blot.  **p < 0.01, ***p < 0.001, and ****p < 0.0001.

Noncontact co‐culture of TAAs with SF295 or SNB19 cells was employed to exclude contact‐dependent cellular communication (Figure 2A). Glioblastoma cells were seeded in the bottom compartment, and the top chamber contained TAAs at a ratio of 1:5 in complete DMEM or DMEM alone for control. After co‐culturing with TAAs for 5 days, TMZ resistance of co‐cultured SNB19 or SF295 cells was evaluated. Flow cytometry analysis showed that the apoptotic rate of SNB19 or SF295 cells after co‐culturing with TAAs decreased obviously after supplementation of TMZ for 48 h (Figure 2C). Besides, when co‐cultured with TAAs for 5 days followed by TMZ exposure for 48 h, the colony formation ability of SNB19 or SF295 cells increased moderately, compared with that of glioblastoma cells without co‐culturing with TAAs (Figure 2D). Furthermore, as a sensor of DNA double‐strand breaks (DSB) induced by TMZ, γ‐H2AX expression lowered obviously in either SNB19 or SF295 cells when co‐cultured with TAAs than that of glioblastoma cells without co‐culturing with TAAs (Figure 2E).

Remarkable high expression of APNG can be observed in SNB19 and SF295 cells at the protein level after co‐culturing with TAAs (Figure 2F), which implies upregulation of APNG expression in glioblastoma cells after co‐culturing with TAAs. Low level of MGMT expression, and no obvious change in SNB19 or SF295 cells after co‐culturing with TAAs (Figure S1B). These findings suggest that APNG expression can inhibit TMZ‐induced apoptosis and conferred TMZ resistance independent of MGMT, thus enhancing TMZ resistance of glioblastoma cells after co‐culturing with TAAs.

### 3.3 APNG knockdown restored TMZ sensitivity of glioblastoma cells

**FIGURE 3:**
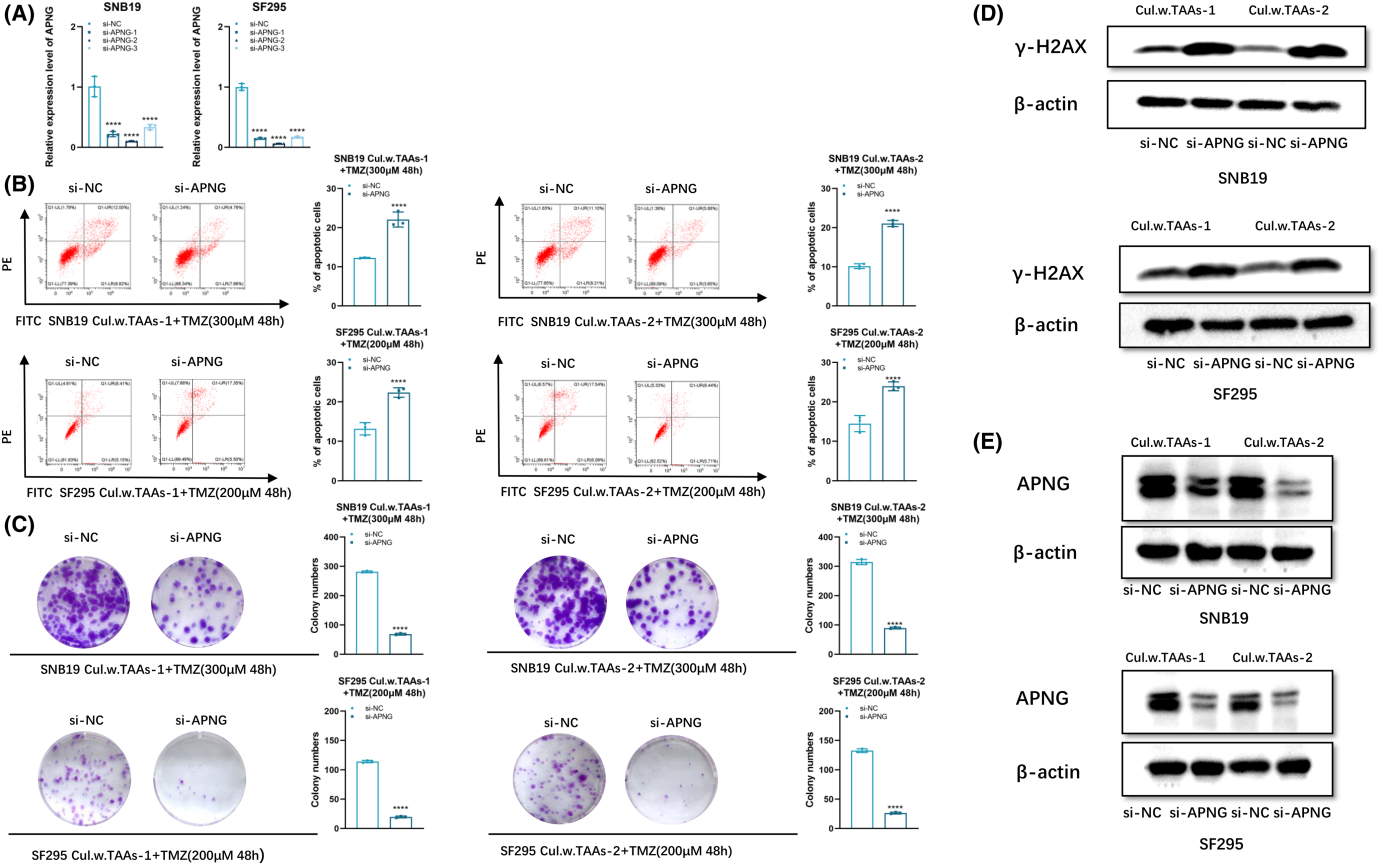
Inhibiting APNG expression promoted TMZ‐induced cell apoptosis. (A) qPCR analysis of APNG expression in SNB19 and SF295 cells with si‐NC, si‐APNG‐1, si‐APNG‐2, si‐APNG‐3 transfection, respectively. (B) Flow cytometry analysis of SNB19 or SF295 cells after co‐culturing with TAAs followed with APNG knockdown and TMZ exposure (300 μM for SNB19, 200 μM for SF295) for 48 h. (C) Soft agar colony formation assay on SNB19 or SF295 cells after co‐culturing with TAAs followed with APNG knockdown and TMZ exposure (300 μM for SNB19, 200 μM for SF295) for 48 h. (D) Western blot of γ‐H2AX expression in SNB19 or SF295 cells after co‐culturing with TAAs followed with APNG knockdown and TMZ exposure (300 μM for SNB19, 200 μM for SF295) for 48 h. (E) APNG protein level of SNB19 or SF295 cells after co‐culturing with TAAs followed with APNG knockdown.  **p < 0.01, ***p < 0.001, and ****p < 0.0001.

For further verifying whether APNG can confer TMZ resistance to glioblastoma cells, three independent APNG siRNAs (si‐APNG‐1, si‐APNG‐2, si‐APNG‐3) were transfected into both SNB19 and SF295 cells, respectively. RT‐PCR verified the effective knockdown of APNG expression of these three siRNAs, and then si‐APNG–2 was selected for the subsequent experiments as it exhibited the strongest knockdown efficiency (Figure 3A).

Flow cytometry disclosed that knockdown of APNG led to cell apoptotic rate increasing in SNB19 and SF295 cells after co‐culturing with TAAs followed by TMZ exposure (Figure 3B). Besides, colony formation assay revealed that clonogenic survival decreased significantly in APNG knockdown SNB19 and SF295 cells co‐cultured with TAAs upon TMZ exposure (Figure 3C). In addition, APNG knockdown resulted in increased accumulation of γ‐H2AX in SNB19 and SF295 cells after co‐culturing with TAAs, compared with the control group upon TMZ administration (Figure 3D). These findings suggest that downregulation of APNG expression enhanced TMZ sensitivity of glioblastoma cells. Besides, after co‐culturing with TAAs, the elevated APNG expression in SNB19 or SF295 cells decreased obviously, when transfected with si‐APNG in glioblastoma cells (Figure 3E).

### 3.4 TAAs‐dependent ALKBH7 delivery increased APNG expression of glioblastoma cells

**FIGURE 4:**
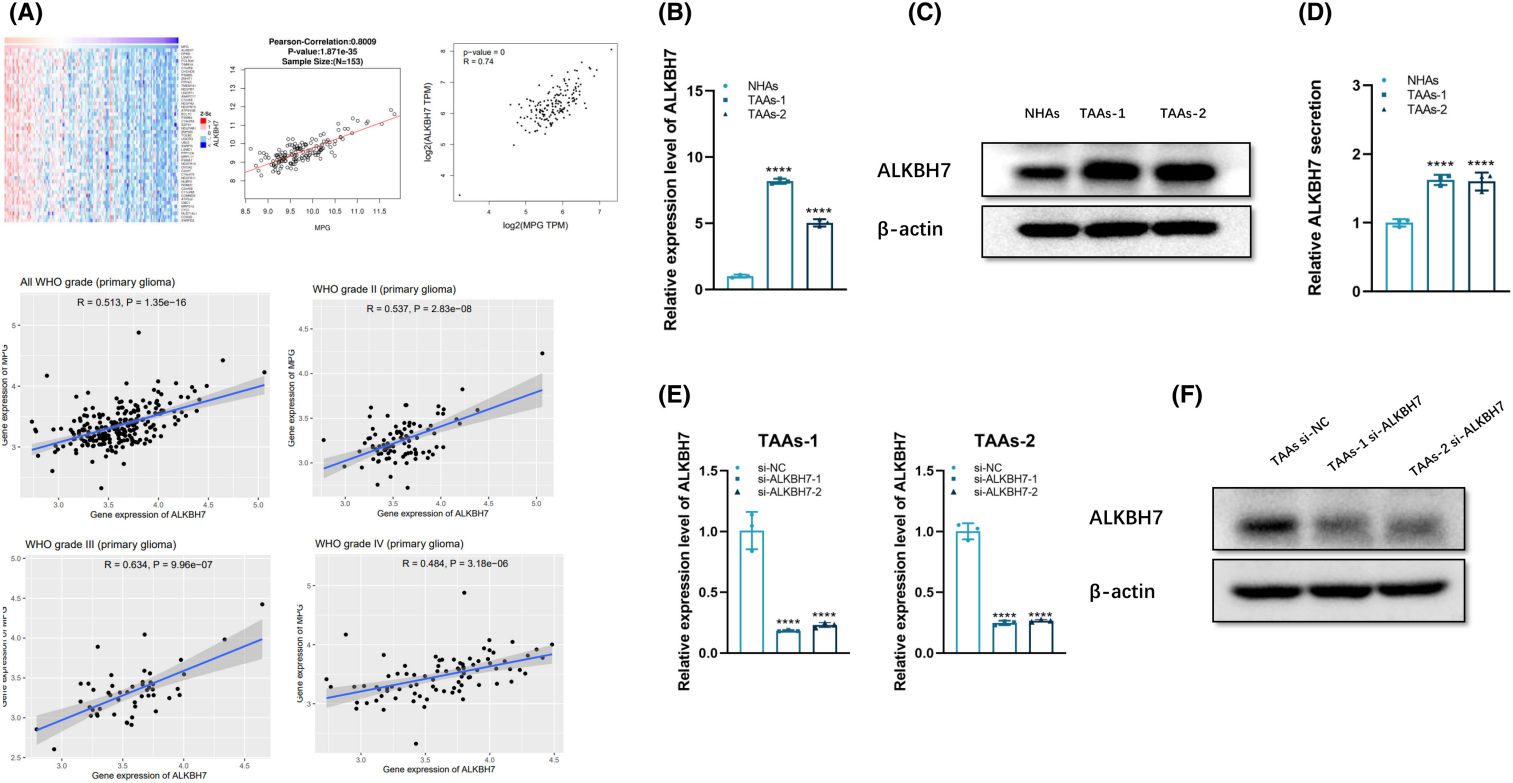
TAAs overexpressed ALKBH7. (A) Bioinformatic assay of the correlation between APNG and ALKBH7 in clinical specimens of glioblastoma. (B) qPCR analysis on ALKBH7 level of TAAs. (C) Western blot on ALKBH7 protein level of TAAs. (D) ELISA for the concentration of secretory ALKBH7 in the culture medium of TAAs, which disclosed higher ALKBH7 level in the culture medium of TAAs than that of NHAs. (E) qPCR of APNG expression in TAAs transfected with si‐NC, si‐ALKBH7‐1 or si‐ALKBH7‐2, respectively. (F) Western blot for ALKBH7 protein level in si‐ALKBH7 TAAs.  **p < 0.01, ***p < 0.001, and ****p < 0.0001.

In order to elucidate the pertinent regulatory mechanisms underlying the heightened expression of APNG in glioblastoma cells subsequent to co‐cultivation with TAAs, bioinformatic prediction was employed based on online analysis of glioma databases. These databases encompassed The Cancer Genome Atlas (TCGA) (https://portal.gdc.cancer.gov), the Chinese Glioma Genome Atlas (CGGA) (http://www.cgga.org.cn), and the Linked Omics Atlas (http://www.linkedomics.org/login.php). By harnessing the capabilities of these extensive repositories, the regulatory molecules pertinent to APNG expression were explored. The ensuing analysis revealed a noteworthy positive correlation between the expression levels of ALKBH7 and APNG (Figure 4A), which provided potential targets for further investigation into the underlying mechanisms driving glioblastoma progression and therapeutic responsiveness.

ALKBH7 expression in NHAs and TAAs was analyzed at both the transcriptional and translational levels, which showed a striking increase of ALKBH7 at the mRNA level in TAAs (Figure 4B) as well as a marked overexpression of ALKBH7 in TAAs at protein level (Figure 4C), suggesting upregulation of ALKBH7 in TAAs. The secretion level of ALKBH7 was detected in the culture medium of TAAs with ELISA, which disclosed secretive ALKBH7 in the culture medium of TAAs was significantly higher than that of NHAs (Figure 4D, Table S1).

To investigate whether TAAs can transfer TMZ resistance to glioblastoma cells through delivering ALKBH7, depletion of ALKBH7 expression in TAAs was performed to evaluate whether TMZ resistance of glioblastoma cells can be restored. For this purpose, two independent ALKBH7 siRNAs (si‐ALKBH7‐1, si‐ALKBH7‐2) were designed and transfected into TAAs, then ALKBH7 expression of si‐ALKBH7 TAAs was detected by RT‐PCR, which confirmed high downregulation efficiency of the two siRNAs (Figure 4E). The si‐ALKBH7‐1 was selected for subsequent experiments as it exhibited higher knockdown efficiency.

**FIGURE 5:**
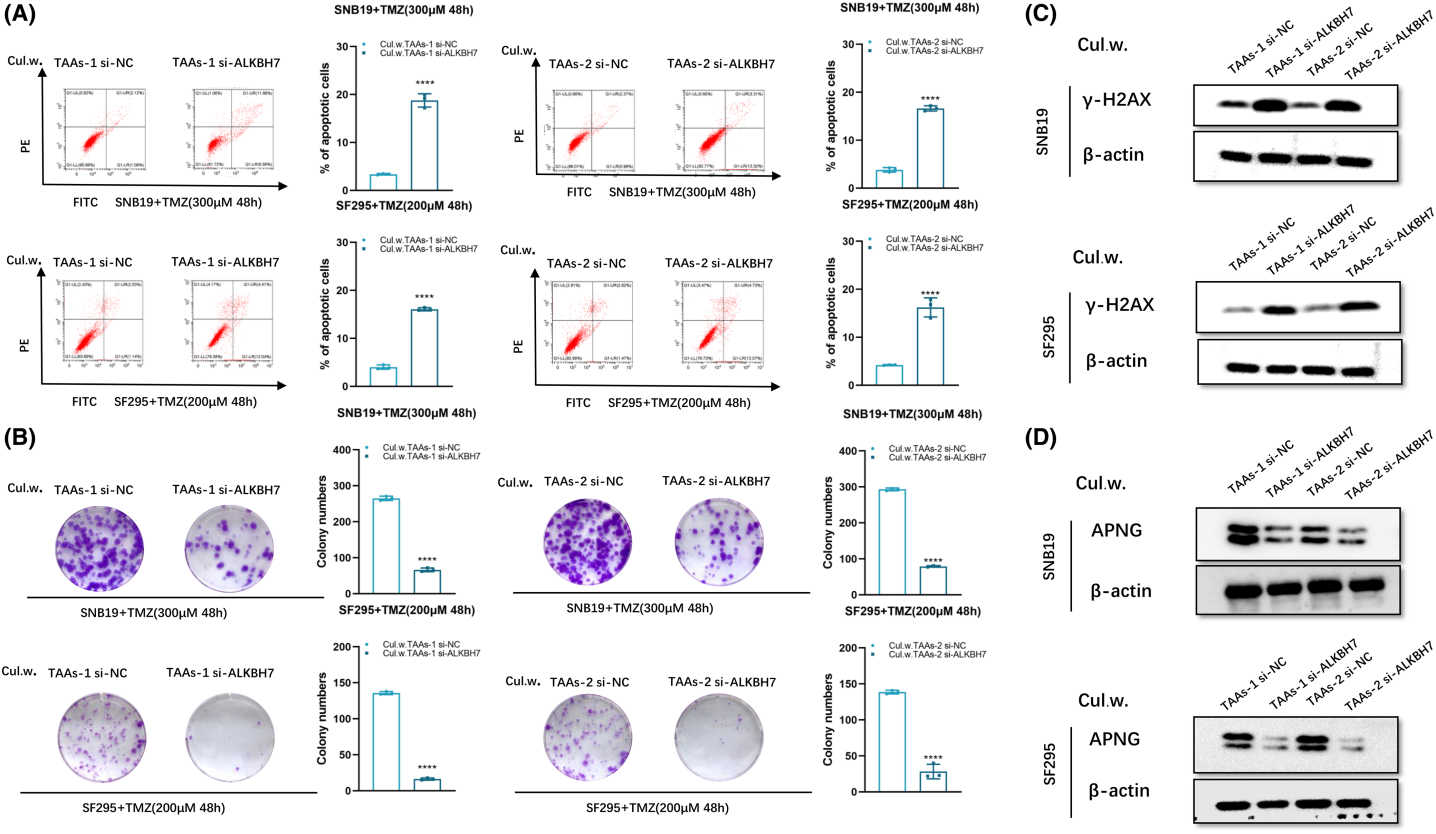
APNG expression of glioblastoma cells decreased after co‐culturing with si‐ALKBH7 TAAs. (A) Flow cytometry on SNB19 or SF295 cells after co‐culturing with si‐ALKBH7 TAAs followed with TMZ exposure (300 μM for SNB19, 200 μM for SF295) for 48 h. (B) Soft agar colony formation assay on SNB19 or SF295 cells after co‐culturing with si‐ALKBH7 TAAs followed with TMZ exposure (300 μM for SNB19, 200 μM for SF295) for 48 h. (C) Western blot of γ‐H2AX expression in SNB19 or SF295 cells after co‐culturing with si‐ALKBH7 TAAs followed with TMZ exposure (300 μM for SNB19, 200 μM for SF295) for 48 h. (D) APNG protein level in SNB19 or SF295 cells after co‐culturing with si‐ALKBH7 TAAs.  **p < 0.01, ***p < 0.001, and ****p < 0.0001.

After knockdown of ALKBH7, the intracellular ALKBH7 level of TAAs declined obviously (Figure 4F). Noncontact co‐culture of glioblastoma cells (SNB19 or SF295, seeded in the bottom compartment) and TAAs, or si‐ALKBH7 TAAs (seeded in the top chamber) was conducted. After co‐culture for 5 days, TMZ resistance of co‐cultured SNB19 or SF295 cells was evaluated, which disclosed that knockdown of ALKBH7 transcription in donor TAAs weakened TMZ‐resistance acquisition of SNB19 or SF295 cells, and apoptotic rate of those two glioblastoma cells increased, as evidenced by flow cytometry after TMZ exposure for 48 h (Figure 5A). Colony formation ability of those two glioblastoma cells decreased after co‐culturing with si‐ALKBH7 TAAs upon TMZ exposure (Figure 5B). Furthermore, a concomitant increase in γ‐H2AX expression of glioblastoma cells can be observed after co‐culturing with si‐ALKBH7 TAAs. Declined transmission of ALKBH7 further indicated that ALKBH7 content of TAAs to transfer TMZ resistance to glioblastoma cells decreased obviously (Figure 5C), which implied that ALKBH7 can regulate APNG expression, knocking‐down of ALKBH7 expression remarkably reduced APNG level (Figure 5D).

### 3.5 ALKBH7‐dependent APNG expression enhanced TMZ resistance of glioblastoma cells

**FIGURE 6:**
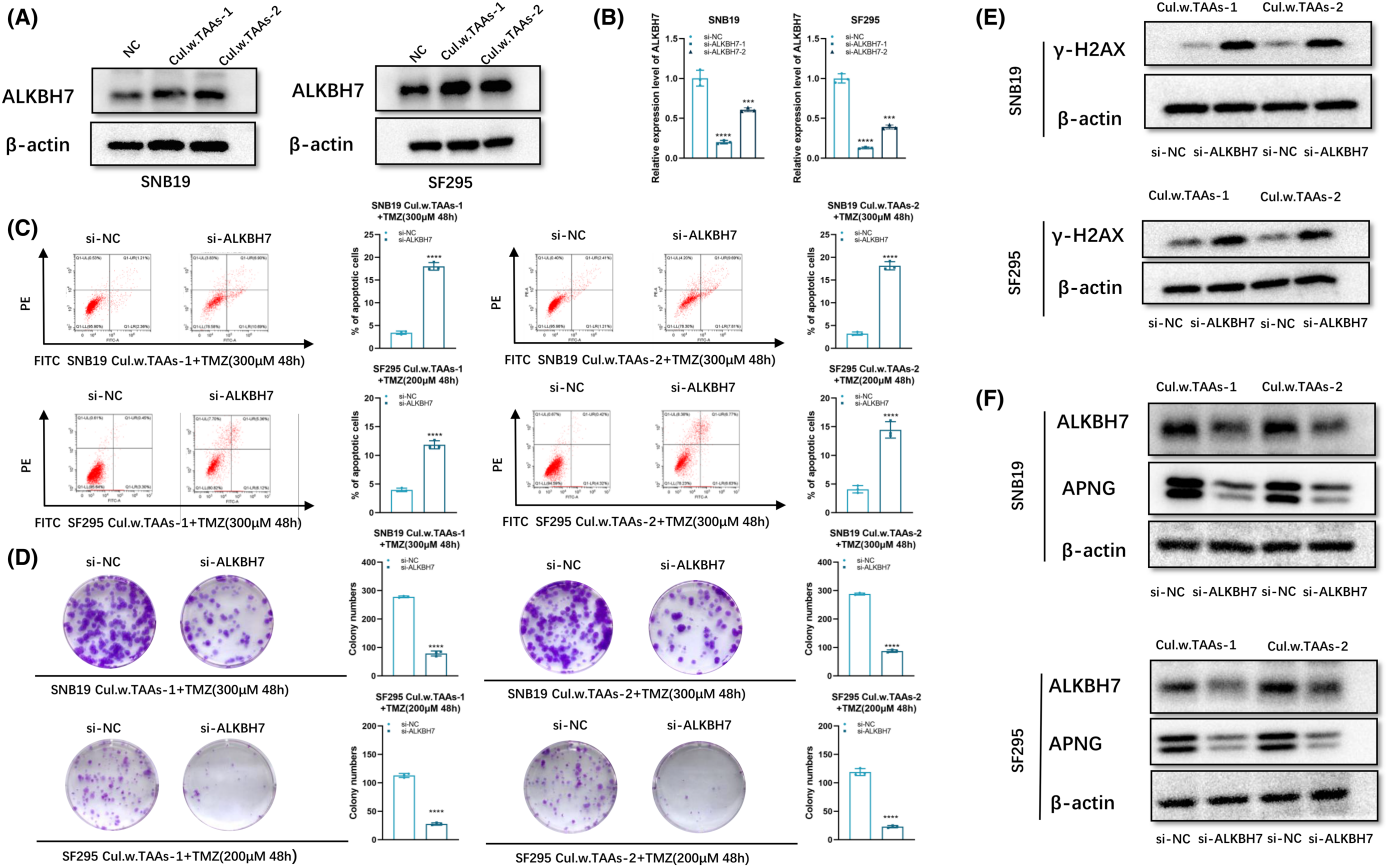
Upregulation of intracellular ALKBH7 increased APNG expression of glioblastoma cells. (A) Western blot for protein level of ALKBH7 in glioblastoma cells after co‐culturing with TAAs. (B) qPCR analysis of ALKBH7 expression in SNB19 or SF295 cells transfected with si‐NC, si‐ALKBH7‐1, or si‐ALKBH7‐2, respectively. (C) Flow cytometry analysis of SNB19 or SF295 cells after co‐culturing with TAAs followed with ALKBH7 knockdown and TMZ exposure (300 μM TMZ for SNB19, 200 μM for SF295 cells) for 48 h. (D) Soft agar colony formation assay of SNB19 or SF295 cells after co‐culturing with TAAs followed with ALKBH7 knockdown and TMZ exposure (300 μM TMZ for SNB19, 200 μM for SF295 cells) for 48 h. (E) Western blot of γ‐H2AX expression of SNB19 or SF295 cells after co‐culturing with TAAs followed with ALKBH7 knockdown and TMZ exposure (300 μM TMZ for SNB19, 200 μM for SF295 cells) for 48 h. (F) The protein level of ALKBH7 and APNG in SNB19 or SF295 cells after co‐culturing with TAAs followed with ALKBH7 knockdown. **p < 0.01, ***p < 0.001, and ****p < 0.0001.

Given the crucial function of APNG on regulating chemo‐resistance of SNB19 and SF295 cells, and MGMT was not involved in these (Figure S1B), the relevance between dramatic alteration of APNG in response to ALKBH7 and glioblastoma chemo‐resistance was further investigated, which disclosed that expression of ALKBH7 in SNB19 and SF295 cells increased after co‐culturing with TAAs (Figure 6A). Two independent ALKBH7 siRNAs (si‐ALKBH7‐1, si‐ALKBH7‐2) were transfected into two glioblastoma cells, respectively. After co‐culturing with TAAs, ALKBH7 expression of either SNB19 or SF295 cells was detected by qRT‐PCR, which showed high efficiency of ALKBH7 knockdown (Figure 6B), and si‐ALKBH7‐1 was selected for subsequent experiments as it exhibited higher knockdown efficiency. Subsequently, glioblastoma cells were co‐cultured with TAAs for 5 days, then transduced with ALKBH7 siRNAs. The apoptotic cells increased significantly in the co‐cultured SNB19 and SF295 cells transfected si‐ALKBH7 after being treated with TMZ for 48 h (Figure 6C). Similarly, colony formation assay revealed that clonogenic survival of glioblastoma cells significantly decreased in si‐ALKBH7 SNB19 and SF295 cells after co‐culturing with TAAs and sequential TMZ exposure (Figure 6D). After co‐culturing with TAAs, the transfer of si‐ALKBH7 into glioblastoma cells resulted in reduced accumulation of γH2AX after TMZ administration (Figure 6E). The protein level of ALKBH7 and APNG was detected by Western blot (Figure 6F), which disclosed that reduced APNG expression in the co‐cultured glioblastoma cells after ALKBH7 knockdown, which implied that APNG expression suppressed cytotoxic effect of TMZ, ALKBH7‐dependent APNG expression was a critical step for TMZ resistance of glioblastoma cells.

### 3.6 ALKBH7 regulated TMZ resistance of glioblastoma cells dependent on APNG expression

It was further investigated whether APNG expression was required in the effect of ALKBH7‐induced TMZ resistance on glioblastoma cells. After co‐culturing with TAAs for 5 days, SNB19 and SF295 were either transfected with si‐ALKBH7 or co‐transfected with si‐ALKBH7&APNG‐oe. Compared with the si‐ALKBH7 group, the apoptosis rate of co‐cultured glioblastoma cells with si‐ALKBH7&APNG‐oe decreased significantly after supplementation of TMZ for 48 h (Figure S2A). In addition, after co‐culturing with TAAs, transfer of si‐ALKBH7&APNG‐oe into glioblastoma cells resulted in reduced accumulation of γ‐H2AX after TMZ administration compared to the si‐ALKBH7 group (Figure S2B). APNG overexpression attenuated the inhibitory effect of ALKBH7 knockdown on TMZ resistance (Figure S2C). Besides, ALKBH7‐knockdown‐mediated TMZ resistance was rescued by APNG overexpression (Figure S2C). Overall, these results demonstrated that ALKBH7 affected TMZ resistance of glioblastoma by regulating APNG expression.

### 3.7 TAAs‐exos confer TMZ resistance to glioblastoma cells

After addition of exosomes derived from NHAs, TAAs, si‐ALKBH7 TAAs or oe‐ALKBH7 TAAs, respectively, SNB19 or SF295 cells were upon TMZ exposure. The results disclosed that the apoptosis rate of glioblastoma cells with addition of TAAs‐exos or oe‐ALKBH7‐exos decreased significantly, compared with NHAs‐exos or si‐ALKBH7 TAAs‐exos group (Figure S3A). In addition, after addition of exosomes derived from NHAs, TAAs, si‐ALKBH7 TAAs or oe‐ALKBH7 TAAs, ALKBH7 expression level of glioblastoma cells with addition of TAAs‐exos or oe‐ALKBH7‐exos increased, compared to those with NHAs‐exos or si‐ALKBH7 TAAs‐exos group (Figure S3B).

### 3.8 ALKBH7 knockdown improved TMZ sensitivity of glioblastoma in vivo

**FIGURE 7:**
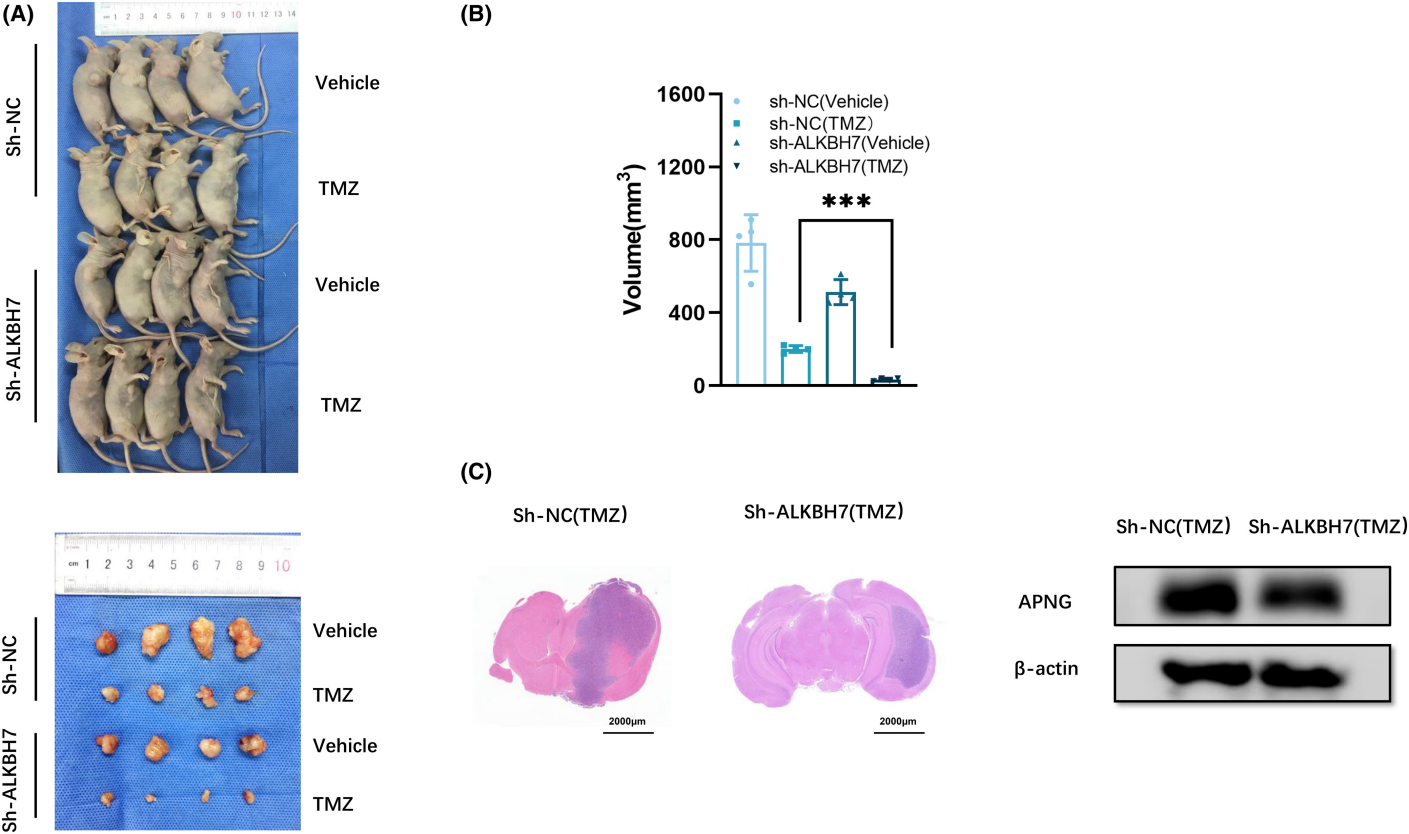
ALKBH7 enhanced chemoresistance of glioblastoma to TMZ in vivo. (A) Subcutaneous xenografts in nude mice derived from sh‐NC or sh‐ALKBH7 SNB19 cells with or without TMZ exposure. (B) The volume of tumor inoculated by sh‐ALKBH7 or sh‐NC SNB19 cells with or without TMZ exposure. ***p < 0.01. (C) Representative H&E staining of brain sections and APNG expression level after intracranial inoculation of sh‐ALKBH7 or sh‐NC SNB19 cells in nude mice treated with TMZ.

ALKBH7 exerted regulatory effects on apoptosis inhibition in APNG‐mediated TMZ resistance of glioblastoma cells, and ALKBH7 delivering via TAAs limited the cytotoxic efficacy of TMZ against glioblastoma cells in vitro. The effect of TAAs‐derived ALKBH7 on TMZ resistance of glioblastoma in vivo was investigated as well. Stable downregulation of ALKBH7 was performed, ALKBH7 depleted (sh‐ALKBH7) or control (sh‐NC) transfected SNB19 cells (1 × 10^6^ cells/per mice) were inoculated into the right axilla of 4‐weeks old Balb/c nude mice through subcutaneous injection (*n* = 16). When the xenografts reached a volume of approximately 50 mm^3^, tumor‐bearing mice received intraperitoneal injections of TMZ (66 mg/kg/day for consecutive 5 days) or an equal volume of DMSO as a control, respectively, 5 days/week, a totally 2 weeks. Stable knockdown of ALKBH7 in glioblastoma cells resulted in smaller xenografts (*p* < 0.01), which implied that ALKBH7 depletion in glioblastoma cells effectively restored the sensitivity of TMZ in vivo (Figure 7A,B). In the intracranial glioblastoma model, downregulation of ALKBH7 in SNB19 cells resulted in less intracerebral tumor proliferation in size and low expression of APNG after concurrent TMZ treatment (Figure 7C), suggesting that downregulation of ALKBH7 increased the therapeutic effect of TMZ in mice against intracranial proliferation of glioblastomas.

## 4 DISCUSSION

Glioblastoma is the most malignant and intractable primary tumor of the central nervous system. Current standard therapies cannot cure, only reach limited survival benefits.^18^ Improving chemotherapeutic efficacy has been a promising way of prolonging patients survival.^19^ As a DNA alkylating agent, and the first‐line chemotherapeutic agent, TMZ has been applied widely to extend patient overall survival in less than 1 year.^20^ However, TMZ resistance is still a major challenge in management of glioblastoma patients, and the underlying mechanisms were under numerous investigations, which were not enough to achieve clinical translation to improve patient outcomes.^21^ Therefore, great necessities are under desire to further investigate the new mechanisms of TMZ resistance and identify novel targets for reversing TMZ resistance of glioblastoma.

Although the impact of astrocytes on glioblastoma progression has been explored previously,^22^, ^23^ however, the influence of astrocytes on glioblastoma development has not been fully clarified. Astrocytes constitute the predominantly abundant glial cell type, accounting for approximately 50% of CNS cells, and function as maintenance of the blood–brain barrier, secretion or absorption of neurotransmitters, as well as regulation of extracellular ion homeostasis.^24^, ^25^, ^26^ Astrocytes can promote tumor cell proliferation by secreting various growth factors to sustain intracranial tumor growth.^27^

The high heterogeneity of glioblastoma was not only based on GSCs and the corresponding progeny tumor cells varied in the diversity of differentiation status, but also multiple intermingling stromal cells in tumor microenvironment (TME), including TAAs.^10^, ^28^ Accumulating evidence has suggested that exosomes (EXOs) can function as important microenvironmental regulators remodeling the phenotypes of various recipient cells, which implies the possibility that GSCs‐derived exosomes might reprogram the phenotypes and the functions of astrocytes in glioblastoma TME. TAAs have been reported to express specific markers, such as TGF‐β and CD44 enhancing glioblastoma invasion, as well as upregulation of CD274 and tenascin‐C to promote the formation of immune suppressive TME,^29^, ^30^, ^31^ indicating the great pro‐tumor potential of TAAs, which derived from transformation of naive astrocytes. Our investigations disclosed that enhanced TGF‐β, CD44, CD274, and tenascin‐C expression can be observed in GSCs‐exos educated astrocytes as well as enhanced proliferation, enlarged cell bodies and more cell processes, suggesting transformation of NHAs to TAAs. TAAs played specific roles in interactions with glioblastoma cells in TME,^32^ they had direct impacts on migration and invasion of glioblastoma cells as well.^33^, ^34^ In vitro co‐culture with astrocytes significantly decreased chemotherapy‐induced apoptosis of A172 glioblastoma cells.^35^ These studies demonstrated both direct interactions between glioblastoma cells and TAAs, as well as indirect crosstalk via secretion of growth factors and cytokines by TAAs can promote glioblastoma development. Furthermore, our studies disclosed a novel mechanism that GSCs‐exos educated TAAs conferred TMZ resistance to glioblastoma cells via delivering ALKBH7 to upregulated APNG.

Exploring new molecular pathways regulating TMZ resistance is fundamental for searching novel therapeutic options that possibly yield improved overall survival.^36^, ^37^, ^38^ In cellular genomic DNA, TMZ yields N7‐meG and N3‐meA alterations, which are well recognized as particularly potentially lethal by blocking the progress of replicative DNA polymerase.^39^ TMZ resistance of glioblastoma cells increased after co‐culturing with TAAs. For O6‐alkylguanine DNA alkyltransferase (MGMT) was one of the most important chemo‐resistance associated protein, we evaluated MGMT expression in glioblastoma cells before and after co‐culturing with TAAs, which disclosed no obvious change (Figure S1B). Therefore, the expression of another drug resistance is associated with protein APNG, which is also closely associated with TMZ resistance of glioblastoma. APNG can fix the cytotoxic lesions N3‐methyladenine and N7‐methylguanine. Numerous studies have shown that APNG can modulate the sensitivity of glioblastoma cells to TMZ and other alkylating drugs, which is independent of O6‐alkylguanine DNA alkyltransferase (MGMT).^40^, ^41^ Since the mutual regulatory relationship existed between ALKBH7 derived from TAAs and APNG expression in glioblastoma cells, which contributed to chemo‐resistance of glioblastoma, targeting ALKBH7 can repress APNG expression and help further increase the antitumor effect of TMZ, which has been verified in the current studies.

ALKBH7 belongs to the AlkB family, which is involved in the processes of alkylation and oxidation‐induced programmed necrosis^42^ and associated with cancer progression.^43^ ALKBH7 was also involved in cellular bioenergetics metabolism,^42^, ^44^ while its full functions have not been fully elucidated. Our studies disclosed its new role of regulating APNG to enhance TMZ resistance of glioblastoma, which deserved further investigation. Besides, APNG was upregulated in glioblastoma cells after co‐culturing with TAAs. TAAs had a high level of ALKBH7 expression, and TAAs‐derived secretory ALKBH7 can induce upregulation of APNG in glioblastoma cells, suggesting the role of ALKBH7 mediating on APNG upregulation during crosstalk between TAAs and glioblastoma cells to promote TMZ resistance. Collectively, our findings emphasized that aberrant activation of the ALKBH7 regulatory network facilitated glioblastoma chemo‐resistance through overexpression of APNG, leading to increased TMZ resistance.

Alkylpurine‐DNA‐N‐glycosylase is a DNA repair enzyme belonging to the base excision repair (BER) system and can repair N3‐methyladenine and N7‐methylguanine adducts produced by alkylating chemotherapeutic agents.^45^, ^46^ APNG had the potential to serve as a therapeutic target and provided a new clue to explain why MGMT‐negative glioblastoma is resistant to TMZ.^40^ Overexpression of APNG was disclosed in several malignancies, including gliomas, breast, and ovarian cancer, which decreased the sensitivity of cancer cells to alkylating agents, especially for TMZ.^47^ In the present study, we observed higher expression of APNG in glioblastoma cells after indirect co‐culturing with TAAs via ALKBH7‐mediated crosstalk, and APNG expression was negatively correlated with the level of DNA damage repair marker γ‐H2AX. Our findings contributed to deeper understanding of the role of ALKBH7 in regulating APNG to promote TMZ resistance during crosstalk between glioblastoma cells and TAAs. The reciprocal regulatory relationship between ALKBH7 and APNG identified in the current study offered a valuable experimental basis for exploring new strategies focusing on ALKBH7‐dependent APNG expression against TMZ‐resistance of glioblastoma.

## 5 CONCLUSIONS

**FIGURE 8:**
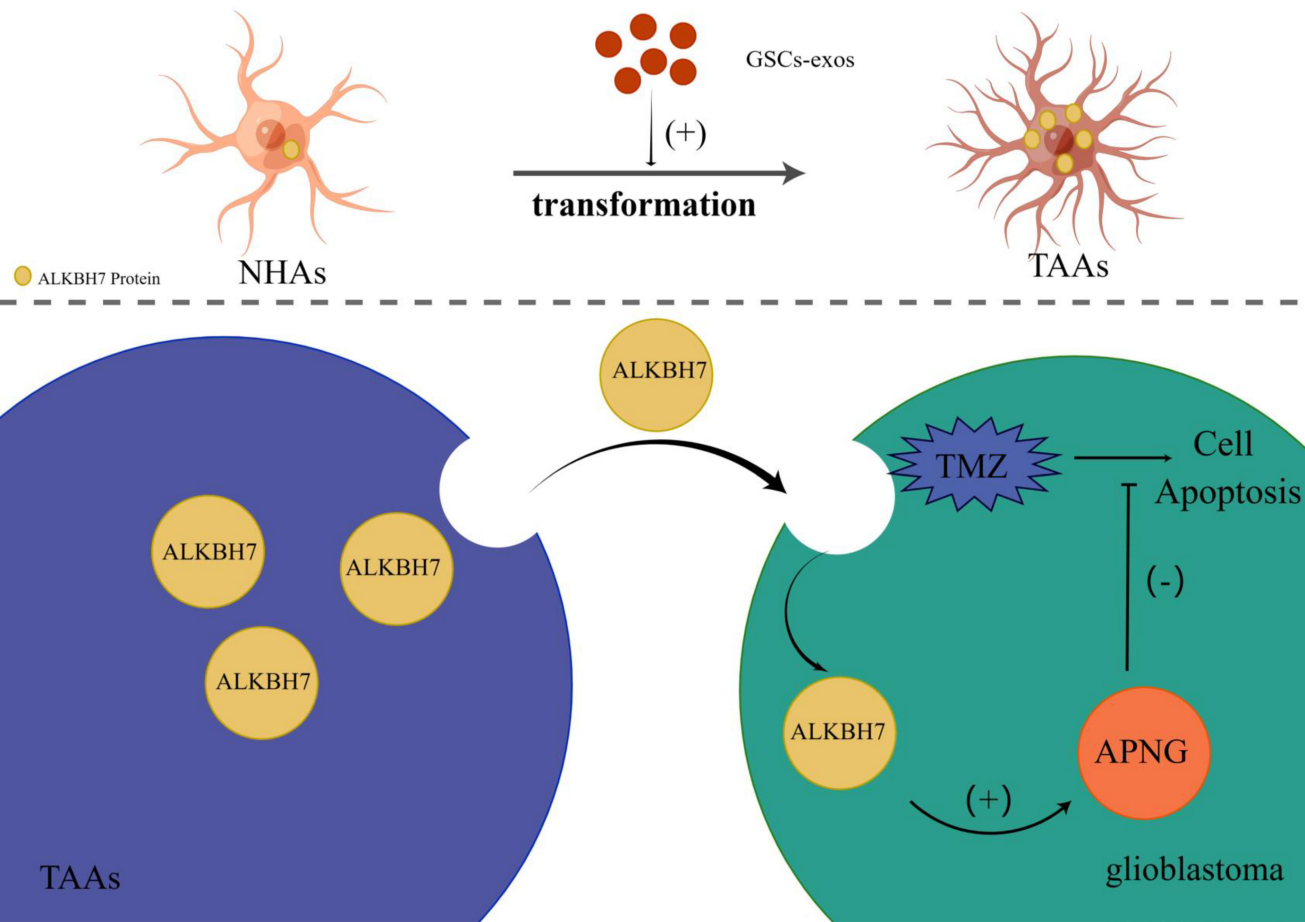
Mechanistic scheme of ALKBH7‐mediated APNG upregulation in enhancing temozolomide resistance of glioblastoma. Molecular mechanism diagram is shown by Figdraw. Schematic of the mechanisms involving ALKBH7 in regulation of TMZ resistance. NHAs transformed to TAAs after the education of GSCs‐exos. Secretory ALKBH7 by TAAs can mediate crosstalk with glioblastoma cells to enhance TMZ resistance of tumor cells via improving APNG expression.

In summary, ALKBH7 was upregulated in GSCs‐exos educated TAAs and can mediate crosstalk with glioblastoma cells leading to upregulation of APNG, thus enhancing chemo‐resistance of glioblastoma. These results further highlighted the ALKBH7/APNG axis contributing to TMZ resistance and provided new evidence that targeting on ALKBH7 might be a potential adjuvant strategy in inhibiting TMZ resistance against glioblastoma (Figure 8).

## AUTHOR CONTRIBUTIONS

LXL performed the conceptualization, methodology validation, and writing—original; LL performed the formal analysis; AYW, SLH, ZPX, XPZ, ZYL, and HRL performed the resources and data curation; JD performed research design and writing—review and editing. All authors read and approved the final manuscript.

## FUNDING INFORMATION

This work was supported by the Jiangsu province key research and development program: Social development project (BE2021653); Key project of Jiangsu Health Commission (ZDB2020016); Natural Science Foundation of Jiangsu Province (BK20201172); Science Foundation of China (82203637).

## CONFLICT OF INTEREST STATEMENT

The authors declare no conflicts of interest.

## CONSENT FOR PUBLICATION

Consents to publish this paper were available from all authors.

## Supporting information


Figures S1–S3

Table S1

## Data Availability

All data generated or analyzed during this study are included either in this article or in the additional files.
