# Supplementary material for: Transformed astrocytes confer temozolomide resistance on glioblastoma via delivering ALKBH7 to enhance APNG expression after educating by glioblastoma stem cells‐derived exosomes
Source: CNS Neurosci Ther. 2024 Feb 8;30(2):e14599. doi: 10.1111/cns.14599 (PMC10853646; doi:10.1111/cns.14599)
Supplement: Supplementary file 1 — Figures S1–S3 [file CNS-30-e14599-s001.docx]

**Supplemental figure.1.**


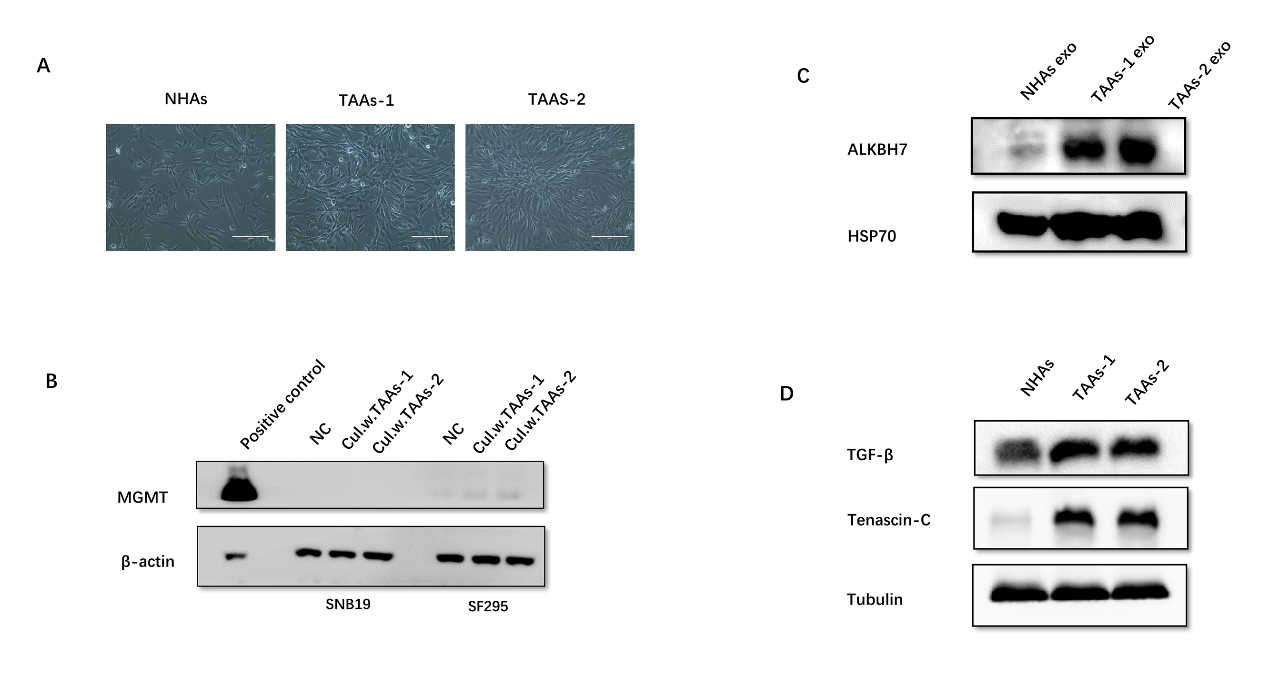


(A). In contrast to NHAs, TAAs had obvious morphological changes larger cell bodies and more longer processes with enhanced proliferation.

(B). Low MGMT expression in both SNB19 and SF295 cells, and no discernible changes at the protein level of MGMT after co-culturing with TAAs.

(C). Western blot on ALKBH7 level in exosomes of NHAs and TAAs

(D). Western blot for TGF-β and Tenascin-C expression in NHAs and TAAs

**Supplemental figure.2. ALKBH7** **regulated the TMZ resistance of GBM cells dependent on APNG expression**


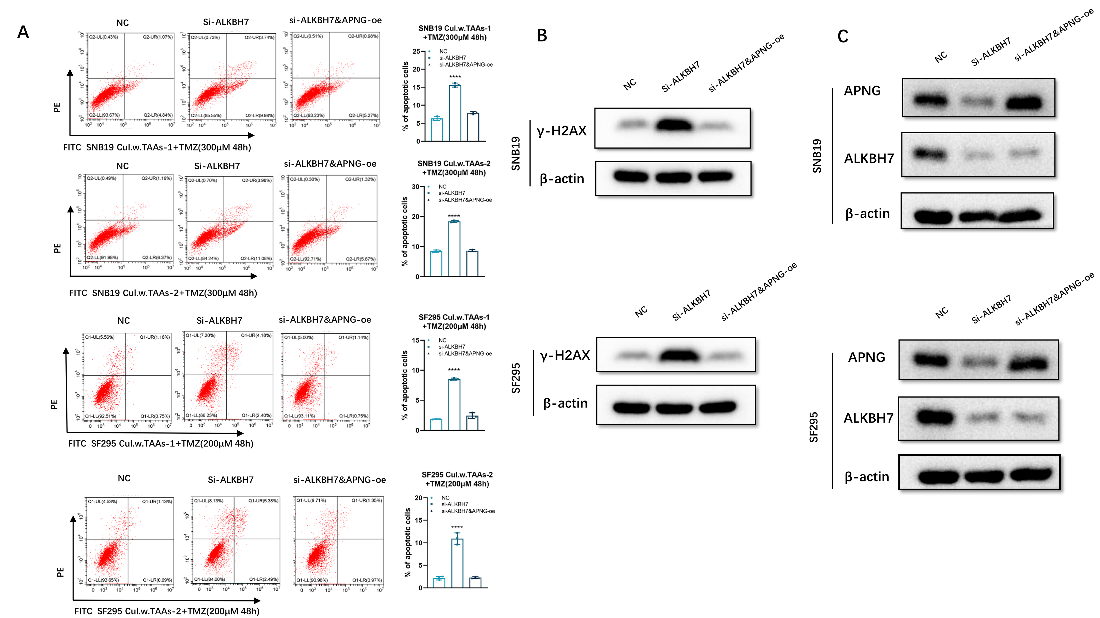


(A) After co-culturing with TAAs for 5 days, SNB19 and SF295 were transfected with si-ALKBH7, or co-transfected with si-ALKBH7&APNG-oe, followed with TMZ exposure (300μM TMZ for SNB19, 200μM for SF295 cells) for 48h, then Flow cytometry analysis of SNB19 or SF295 cells were performed

(B) After co-culturing with TAAs for 5 days, SNB19 and SF295 were transfected with si-ALKBH7, or co-transfected with si-ALKBH7&APNG-oe, followed with TMZ exposure (300μM TMZ for SNB19, 200μM for SF295 cells) for 48h, then Western blot analysis of γ-H2AX expression in SNB19 or SF295 cells were performed.

(C) After co-culturing with TAAs for 5 days, SNB19 and SF295 were transfected with si-ALKBH7, or co-transfected with si-ALKBH7&APNG-oe, then ALKBH7 and APNG expression of SNB19 or SF295 cells by WB was performed.

The quantitative data are presented as means ± SEM of biological triplicate experiments (*P < 0.05, **P < 0.01 and ***P < 0.001).

**Supplemental figure.3 TAAs-exos confer TMZ resistance to glioblastoma cells**


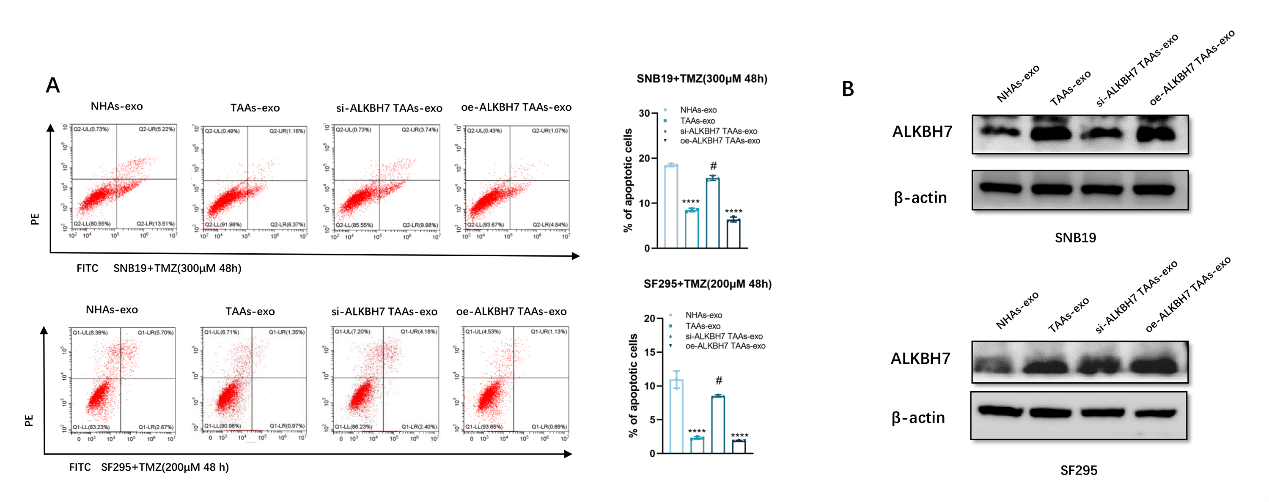


（A）Apoptotic rate of SNB19 or SF295 cells after co-cultured with exosomes derived from NHAs, TAAs, si-ALKBH7 TAAs or oe-ALKBH7 TAAs, respectively, followed with TMZ exposure, which disclosed lower apoptotic cells in glioblastoma cells with TAAs-exos or oe-ALKBH7-exos, compared to those with NHAs-exos or si-ALKBH7 TAAs-exos.

(B) ALKBH7 expression after co-cultured with exosomes derived from NHAs, TAAs, si-ALKBH7 TAAs or oe-ALKBH7 TAAs, respectively, which disclosed higher expression of ALKBH7 in glioblastoma cells with TAAs-exos or oe-ALKBH7-exos, compared to those with NHAs-exos or si-ALKBH7 TAAs-exos.
