# Supplementary material for: Transformed astrocytes confer temozolomide resistance on glioblastoma via delivering ALKBH7 to enhance APNG expression after educating by glioblastoma stem cells‐derived exosomes
Source: CNS Neurosci Ther. 2024 Feb 8;30(2):e14599. doi: 10.1111/cns.14599 (PMC10853646; doi:10.1111/cns.14599)
Supplement: Supplementary file 2 — Table S1 [file CNS-30-e14599-s002.docx]

|  | NHAs | TAAs-1 | TAAs-2 |
| --- | --- | --- | --- |
| ELASA concentration（pg/ml） | 584 | 1410 | 1327 |
|  | 623 | 1532 | 1540 |
|  | 916 | 1540 | 1513 |

Table S1: Caption Text: The ALKBH7 content in the supernatant of NHAs, TAAs-1, and TAAs-2 cells, as determined by ELISA."
